# Reproductive performance and gestational effort in relation to dietary fatty acids in guinea pigs

**DOI:** 10.1186/s40104-017-0158-4

**Published:** 2017-04-01

**Authors:** Matthias Nemeth, Eva Millesi, Carina Siutz, Karl-Heinz Wagner, Ruth Quint, Bernard Wallner

**Affiliations:** 1 Department of Behavioural Biology, University of Vienna, Althanstrasse 14, 1090 Vienna, Austria; 2 Department of Nutritional Sciences, University of Vienna, Althanstrasse 14, 1090 Vienna, Austria; 3 Department of Anthropology, University of Vienna, Althanstrasse 14, 1090 Vienna, Austria

**Keywords:** Body mass, Female reproduction, Gestation, Litter size, Polyunsaturated fatty acid, Saturated fatty acid, Total litter mass

## Abstract

**Background:**

Dietary saturated (SFAs) and polyunsaturated (PUFAs) fatty acids can highly affect reproductive functions by providing additional energy, modulating the biochemical properties of tissues, and hormone secretions. In precocial mammals such as domestic guinea pigs the offspring is born highly developed. Gestation might be the most critical reproductive period in this species and dietary fatty acids may profoundly influence the gestational effort. We therefore determined the hormonal status at conception, the reproductive success, and body mass changes during gestation in guinea pigs maintained on diets high in PUFAs or SFAs, or a control diet.

**Results:**

The diets significantly affected the females’ plasma fatty acid status at conception, while cortisol and estrogen levels did not differ among groups. SFA females exhibited a significantly lower body mass and litter size, while the individual birth mass of pups did not differ among groups and a general higher pup mortality rate in larger litters was diminished by PUFAs and SFAs. The gestational effort, determined by a mother’s body mass gain during gestation, increased with total litter mass, whereas this increase was lowest in SFA and highest in PUFA individuals. The mother’s body mass after parturition did not differ among groups and was positively affected by the total litter mass in PUFA females.

**Conclusions:**

While SFAs reduce the litter size, but also the gestational effort as a consequence, PUFA supplementation may contribute to an adjustment of energy accumulations to the total litter mass, which may both favor a mother’s body condition at parturition and perhaps increase the offspring survival at birth.

**Electronic supplementary material:**

The online version of this article (doi:10.1186/s40104-017-0158-4) contains supplementary material, which is available to authorized users.

## Background

Reproduction represents the energetically most demanding life history stage in mammalian females. Adequate and balanced dietary intakes of specific macronutrients are of major importance to ensure an appropriate energy supply for maintaining reproductive performances [1]. In this context, dietary fatty acids have been suggested to play a major role in providing energy for an organism as well as by affecting hormone secretions and cell membrane functions in the central nervous system and the reproductive tract. The dietary fatty acid content and composition, particularly the amounts and ratios of specific types of fatty acids in the diet, can therefore directly modulate the physiology of reproduction and ultimately an individual’s reproductive success [2, 3].

Among dietary fat types, omega-3 (n-3) and omega-6 (n-6) polyunsaturated fatty acids (PUFAs), including the essential α-linolenic acid (ALA, 18:3 n-3) and linoleic acid (LA, 18:2 n-6) and their long-chain metabolites eicosapentaenoic acid (EPA, 20:5 n-3), docosahexaenoic acid (DHA, 22:6 n-3), and arachidonic acid (AA, 20:4 n-6), can positively influence reproductive processes in females. Diets high in ALA or in the n-3 long-chain metabolites EPA and DHA have been shown to promote ovulation and increase the number of released ova in rats [4], and may further increase conception rates and reduce pregnancy losses in cows [5]. PUFAs in general, including ALA and LA, can also promote the prenatal development of rabbits and mice, resulting in increased body mass and improved physical condition at birth [6, 7]. Although less well studied, dietary intakes of non-essential saturated fatty acids (SFAs) can also improve reproductive performances by increasing the birth mass in rats [8] or the sex ratio at birth towards more male offspring in mice [9]. However, dietary SFAs may not only promote reproductive functions as PUFAs obviously do, since various effects on metabolic processes may be detrimental for an individual [10, 11]. The effects of dietary fatty acids may be simply explained by their high energy content and therefore increased energy allocation for reproductive functions and prenatal development, or by improving a mother’s body condition and hormone secretion rates already at the time of conception [3].

By modulating litter size, sex ratio, and the offspring’s birth mass, dietary fatty acids not only affect the reproductive output, but also a female’s body mass change during pregnancy, reflecting the gestational effort. Especially in precocial mammalian species such as the domestic guinea pig (*Cavia aperea f. porcellus*) pups are born highly developed and gestation is basically characterized by a very high investment of mothers [12]. Larger litter sizes obviously cause higher gestational efforts in guinea pigs and are usually characterized by a lower birth mass of single pups and a higher rate of stillbirths compared to smaller litters [13, 14]. Although lactation in guinea pigs definitely represents an important and energetically demanding period, this may be less pronounced compared to altricial mammalian species. The prolonged gestation period can be assumed to be the energetically more demanding reproductive period in guinea pigs, especially as newborn pups are of relatively high body mass and the lactation period occurs to be rather short [15, 16]. Since mortality in term-born guinea pig pups is relatively low [17], probably due to the high developmental stage at birth, reproduction in guinea pigs can be considered as highly efficient and could probably be further promoted by supplementations with dietary fatty acids.

The aim of this study was therefore to determine and compare the effects of diets high in PUFAs or SFAs on reproductive output, offspring survival and condition, and body mass changes in female guinea pigs during gestation. Due to the relatively long gestation period and the precociality, guinea pigs may represent an adequate model species to study the effects of dietary fatty acids during gestation and prenatal development. Knowledge on such influences may further increase the understanding for the relevance of dietary fatty acids regarding an individual’s reproductive success in general, but also how these nutrients may influence the process of gestation in mothers in relation to the offspring’s prenatal development.

## Methods

### Ethical statement

Experiments were conducted in accordance with EU Directive 2010/63/EU for animal experiments and the Austrian laws for animal experiments and animal keeping. The study has been checked and approved by the internal board on animal ethics and experimentation of the Faculty of Life Sciences of the University of Vienna (# 2014–005) and the Austrian Federal Ministry of Science and Research (BMWF-66.006/0024-II/3b/2013).

### Animals and housing conditions

All domestic guinea pigs (30 males and 30 females) used for this study were bred at the Department of Behavioural Biology at the University of Vienna. All animals were adult, sexually intact, and accustomed to the daily contact with humans. Differences in natural fur colorations allowed an individual identification. Animals were housed in single-sexed groups of ten individuals, resulting in three male and three female groups. Each group’s enclosure (2 m × 1.6 m) was environmentally enriched with shelters and platforms and the floor was covered with standard bedding material. Animals were housed at a temperature of 20 ± 2 °C, 50 ± 5% humidity, and a light-dark cycle of 12 h with lights on at 0700 h.

The daily provided food consisted of guinea pig pellets (ssniff V2233, ssniff Spezialdiäten GmbH, Soest, Germany) and 50 g of hay per group; for precise nutrient composition of guinea pig pellets see [18]. According to the manufacturer’s information, guinea pig pellets contain 3% (w/w) crude fat, which accounts for 8% of the convertible energy provided with the food (gross energy content of guinea pig pellets: 16.6 MJ/kg; see Table 1 for fatty acid composition of the guinea pig pellets). Water was provided in several drinking bottles. In addition to the daily provided standard food, each male and female group received one of three different dietary supplements: walnut oil (high in PUFAs; gross energy content: 38.7 MJ/kg, fat content: 99.6%), coconut fat (high in SFAs; gross energy content: 38.9 MJ/kg, fat content: 100%), or pure water in case of a control group (see Table 1 for fatty acid compositions of walnut oil and coconut fat). Each animal was administered orally with 3 mL of the specific supplement per 1 kg body mass on every day of the experiment using 1 mL syringes. This procedure, including the supplemented amounts, has already been applied previously in studies on the effects of dietary fatty acids in rats and guinea pigs and proved successful in affecting the fatty acid status of the individuals [19, 20] and result approximately in 25% energy intake from dietary fats in these animals. An imbalance in the total energy intake among single individuals and the three dietary groups, which would have been caused by the additional fatty acid supplementations, was counteracted by ad libitum feeding of guinea pig pellets.

**Table 1:** Most prominent fatty acids (% of total fatty acids based on gas chromatography analyses) of guinea pig pellets, walnut oil, and coconut fat

Fatty acid	Guinea pig pellets^a^	Walnut oil	Coconut fat
C 12:0	n.d.	n.d.	49.26
C 14:0	0.61	n.d.	22.53
C 16:0	16.16	6.55	11.82
C 16:1	0.61	n.d.	n.d.
C 18:0	3.35	2.76	4.60
C 18:1 n-9	18.90	14.16	6.71
C 18:1 n-7	n.d.	1.28	0.53
C 18:2 n-6	50.00	63.14	3.73
C 18:3 n-6	n.d.	0.40	0.26
C 18:3 n-3	10.06	11.03	n.d.
total MUFAs	19.51	15.44	7.39
total PUFAs	60.06	75.25	4.22
total SFAs	20.43	9.31	88.39
P:S ratio	2.94	8.08	0.05

*n.d.* Not detectable

^a^Ssniff V2233, ssniff Spezialdiäten GmbH, Soest, Germany

Guinea pig pellets were provided ad libitum in each dietary group; walnut oil (PUFA group) and coconut fat (SFA group) were additionally supplemented (3 mL/kg body mass) to animals of the corresponding group

### Experimental procedure

Animals of the different groups were compared in their age and body mass in advance to the experiment to exclude possible differences in these variables at the onset of the study. Males were included in these pre-experimental analyses in order to exclude any possible influences of male body condition on female reproductive performance. Using the statistical package R 3.2.2 [21] and two-way analyses of variance, no differences among the dietary and/or sex groups were found in age (*F*_5,54_ = 0.566, *P* = 0.726; mean age: 21.1 ± 1.2 month) or body mass (*F*_5,54_ = 0.746, *P* = 0.593; mean body mass: 805 ± 20 g) at the beginning of the experiment.

The experimental procedure started with an initial 100-d feeding phase. All animals were weighed daily at 0900 h and the body mass-based supplementations were carried out, which lasted no longer than 1 min in total per animal. The dietary supplementations for 100 d should ensure that the individuals had highest possible levels of specific fatty acids and a maximum incorporation into neuronal membranes and tissues of the reproductive tracts. After d 100, blood samples were collected from all animals to measure plasma fatty acid levels as indicators of the general fatty acid status [22, 23] and females were mated with heterogeneous males maintained on the same dietary supplements as mentioned above. For this purpose, male and female groups, supplemented with the same fatty acids, were randomly mixed in two mating groups, each consisting of five males and five females. This yielded a total of six mating groups, two for each dietary regime. Mating in small groups with a sex ratio of 1:1 should reduce male competition for females and increase the number of social partners in order to reduce social stress [24] and, therefore, reliably result in frequent pregnancy rates. During the mating period, saliva and plasma samples were collected from females to analyze saliva cortisol and plasma estrogen levels at conception. Both hormones served as indicators of female homeostasis at conception. Once pregnancy was detected, the respective female was removed from the mating group and introduced to its single-sexed group again. Weighing and dietary supplementation procedures continued throughout the experiment and were still carried out daily at 0900 h. until the first day after parturition.

### Measurements of gestational and reproductive performance

During mating, the vaginal membrane of each female was inspected visually to monitor receptivity during the estrous cycle, because the vagina appears to be opened at this stage of proestrus/estrus for 1–6 d [25]. To define the day of conception, a time span of approximately 66–69 d was counted back from the day of parturition. This constant period of gestation is well documented in guinea pigs (see for example [26]). The first day with an opened vagina within this time frame represented the day of conception. Due to a relatively long estrous cycle in guinea pigs of about 16 d [27], a misinterpretation was very unlikely, as this would have resulted in unnaturally short (~50 d) or long (~85 d) gestation periods. The number of days from conception to parturition was defined as gestation duration.

Most females gave birth during the night and therefore the offspring of each female was counted, sexed, and weighed 12 h after parturition at the latest. Also pups that were found dead, but fully developed, were weighed and sexed. The body mass of mothers at conception, during gestation, on the last day of gestation (1 d before parturition), and after parturition was recorded using a standard electronic scale (accuracy ± 1 g) to monitor changes in body mass throughout gestation and parturition.

### Saliva and blood sampling procedures

Saliva samples were collected by inserting standard cotton buds into the animal’s mouth for approximately 1 min. Cotton buds were then centrifuged (14,000 rpm, 10 min) and pure saliva was stored at −20 °C until further analysis. Blood was collected with heparinized micropipettes after punctuation of prominent ear veins. Plasma was separated by centrifugation (14,000 rpm, 10 min) and stored at −20 °C until further analysis (for further information regarding sample collection procedures see [28]).

### Hormone analyses

Hormone concentrations in saliva and plasma were analyzed by biotin-strepdavidin enzyme-linked immunoassays [29, 30]. Saliva samples were diluted 1:50 and cortisol concentrations measured in 10 μL aliquots using a cortisol-specific antibody. Extraction of plasma hormones was done by adding 2 mL diethylether to 100 μL plasma, shaking the samples four times for 15 min, and freezing them overnight. After evaporation of the diethylether (30 °C, 10 min), samples were diluted 1:4 and concentrations of total estrogens in plasma were determined in 25 μL aliquots using an antibody against total estrogens. For further information regarding the used antibodies, including cross- reactions with relevant steroids, see Palme & Möstl [29, 30]. All analyses were run in duplicates. The confidence criterion was set at ≤ 15% for the coefficient of variance of the sample duplicates. Intraassay coefficients of variance for saliva cortisol and plasma estrogen concentrations were 11.15 and 8.64%, respectively.

### Plasma fatty acid analyses

Proportions of fatty acids in plasma prior to mating and the gestational period were analyzed by gas chromatography. Following the protocols by Wagner et al. [31] and Nemeth et al. [22], fatty acids in 35 μL plasma were transesterificated by adding 1 mL methanolic NaOH, containing butylated hydroxytoluene, and 1 mL boron-trifluoride to obtain fatty acid methyl esters (FAMES). Samples were boiled for 5 min at 100 °C and cooled on ice for 10 min after each step. FAMES were then extracted by adding 500 μL hexane four times, including 5 min of shaking the samples (700 rpm) in between each addition. Samples were evaporated at 40 °C under nitrogen and redissolved in hexane. FAMES were separated by an Auto-System-Gaschromatograph (Perkin Elmer, USA) with flame ionization detector, equipped with an Rtx-2330 30 m × 0.25 mm × 0.20 μm silica column. 1 μL of prepared samples was injected under a 1:25 split at 250 °C and detected at 275 °C; helium was used as carrier gas. Fatty acids were identified using a 37 component FAME Mix Standard (Supelco, Bellafonte, USA) and TotalChrome Workstation 6.3.0 (PE Nelson, Perkin Elmer, USA) was used for peak integration. Fatty acids are expressed as percentage of total fatty acids.

### Statistical analyses

Statistical analyses were carried out using R version 3.2.2 [21] and the implemented package ‘nlme’ [32] for performing linear mixed effect models (LMEs). For post-hoc analyses, the packages ‘phia’ [33] and ‘PMCMR’ [34] were used. Package ‘effects’ [35] was used to extract effect plots.

Based on the distribution of the data, conditions at conception and reproductive parameters were analyzed and compared among groups (control, PUFA, SFA) using one-way analyses of variance (ANOVAs), Kruskal-Wallis, or Pearson’s Chi-squared tests. The sex ratio and survival rate at birth were analyzed by generalized linear models (GLMs) with binomial link. Single pup’s birth mass was analyzed using LMEs, including ‘group’, ‘sex’, and their interaction as fixed effects, and ‘mother’ as random effect to correct for the relatedness. To control for possible litter size effects, ‘litter size’ was included in the models as covariate.

The body mass during gestation was analyzed by an LME, including ‘group’ (control, PUFA, SFA), ‘day’ as second-order polynomial term, and ‘total litter mass’, as well as their interactions as fixed effects, and ‘individual ID’ as random effect to correct for repeated measurements. The second-order polynomial of ‘day’ was also included as random slope to correct for individual changes in body mass during gestation. In the beginning, models including either ‘total litter mass’ or ‘litter size’ as covariate were compared based on the Akaike information criterion (AIC). This analysis suggested to include total litter mass rather than litter size as fixed effect, although litter size is usually used as covariate to analyze the body mass during gestation (see for example [26]). Total litter mass was also preferred due to the relatively low variation of litter size in the SFA group and should definitely represent the litter size effect, as litter size and total litter mass were highly related (R^2^ = 0.87, *P* = 3 × 10^−13^).

To analyze the gestational effort, body mass in females prior to parturition and on the first day after parturition were first compared among groups by ANOVAs. Afterwards, linear models were calculated, including ‘group’ (control, PUFA, SFA), ‘body mass at conception’, and ‘total litter mass’, as well as their interactions as predictor variables, therefore reflecting the gestational effort as the body mass increase during gestation corrected for the body mass at conception.

Model assumptions (linearity, normality, homoscedasticity of residuals) were checked by performing Shapiro-Wilk normality tests and Levene’s test for homogeneity of variance as well as by model diagnostic plots of residuals and fitted values. Models were fitted (removal of non-relevant interaction and main effects) based on the AIC. Only the highest significant interaction and/or main effects are considered in the result section. Model statistics are based on type 3 sum of squares. All post-hoc analyses were Bonferroni corrected. The level of significance was set at *P* ≤ 0.05.

## Results

### Plasma fatty acids

The dietary treatments resulted in different plasma fatty acid proportions among the female groups (Table 2). According to the diets, SFA females showed highest percentages in total SFAs, while PUFA females showed lowest percentages in all types of SFAs. PUFA females, in contrast, showed highest percentages in n-3 and n-6 PUFAs, while n-9 MUFAs were highest in control females. The plasma n-6:n-3 ratio was significantly higher in SFA females, while PUFA females showed the highest P:S ratio.

**Table 2:** Most prominent plasma fatty acids (% of total plasma fatty acids) in female guinea pigs maintained on a control, high-PUFA, or high-SFA diet

Fatty acid	Control	PUFA	SFA	*F*-value	*P*-value
C 12:0	0.06 ± 0.02^a^	0.07 ± 0.02^a^	1.27 ± 0.36^b^	30.059	<0.001
C 14:0	0.93 ± 0.08^a^	0.59 ± 0.04^a^	4.64 ± 0.61^b^	88.240	<0.001
C 16:0	17.12 ± 0.23^a^	12.70 ± 0.39^b^	16.80 ± 0.49^a^	40.270	<0.001
C 18:0	10.09 ± 0.52^a^	6.84 ± 0.41^b^	9.05 ± 0.40^a^	113.780	<0.001
C 18.1 n9	15.53 ± 0.46^a^	12.85 ± 0.70^b^	12.49 ± 0.59^b^	7.901	0.002
C 18:2 n6	39.77 ± 1.00^a^	49.13 ± 0.86^b^	41.73 ± 0.60^a^	34.695	<0.001
C 18:3 n3	6.02 ± 0.22^a^	7.71 ± 0.14^b^	4.47 ± 0.36^c^	19.890	<0.001
total n-9	16.21 ± 0.47^a^	15.42 ± 0.46^a^	13.13 ± 0.58^b^	9.991	<0.001
total n-6	43.09 ± 0.93^a^	51.41 ± 0.90^b^	44.62 ± 0.67^a^	27.577	<0.001
total n-3	6.64 ± 0.24^a^	8.12 ± 0.45^b^	4.95 ± 0.36^c^	19.400	<0.001
total SFA	31.67 ± 0.74^a^	23.74 ± 0.91^b^	35.31 ± 0.81^c^	51.931	<0.001
total MUFA	18.51 ± 0.50^a^	16.66 ± 0.49^b^	15.03 ± 0.63^b^	10.111	<0.001
total PUFA	49.82 ± 1.08^a^	59.60 ± 0.89^b^	49.66 ± 0.55^a^	43.030	<0.001
n-6 : n-3 ratio	6.55 ± 0.22^a^	6.57 ± 0.51^a^	9.59 ± 0.88^b^	9.254	<0.001
P : S ratio	1.59 ± 0.07^a^	2.56 ± 0.13^b^	1.42 ± 0.04^a^	50.46	<0.001

Different superscript letters indicate significant differences between single groups (*P* ≤ 0.05)

### Body conditions at conception

The body mass at conception, approximately 100 d after the onset of the feeding procedure, differed among the groups (*F*_2,25_ = 6.954, *P* = 0.004) and was significantly lower in SFA females compared to control females (control: 942 ± 22 g; PUFA: 938 ± 50 g; SFA: 841 ± 15 g; control-PUFA: *χ* ^*2*^ = 0.006, *P* = 1.000; control-SFA: *χ* ^*2*^ = 13.998, *P* = 0.001; PUFA-SFA: *χ* ^*2*^ = 3.447, *P* = 0.190). No differences were detected in saliva cortisol concentrations (*F*_2,25_ = 1.891, *P* = 0.172; control: 18.77 ± 4.45 ng/mL; PUFA: 58.97 ± 23.81 ng/mL; SFA: 40.24 ± 12.68 ng/mL) or plasma estrogen levels (*F*_2,17_ = 2.472, *P* = 0.114; control: 0.48 ± 0.09 ng/mL; PUFA: 0.39 ± 0.08 ng/mL; SFA: 0.24 ± 0.07 ng/mL) at conception.

### Overall reproductive output

A total of 28 female guinea pigs became pregnant and showed successful gestations, resulting in normal parturitions at term. Most females became pregnant in their first estrus during the mating phase, with no differences among groups in the number of estrous cycles until conception (*χ* ^*2*^ = 2.505, *P* = 0.286). A total of 85 pups were born fully developed (52 ♂ : 33 ♀). Except for a total litter loss of five pups at birth in one control female, the number of fully-developed, but dead-born pups was very low (71 alive : 14 dead). All dead-born pups were found in their amniotic sacs. The overall reproductive output, including pregnancy rates, total number of pups, total sex ratio, and ratio of alive and dead born pups, did not differ among the groups (Table 3).

**Table 3:** Statistical analysis of the overall reproductive output for groups of female guinea pigs (*n* = 10 per group) maintained on a control, high-PUFA, or high-SFA diet

Reproductive parameter	Control	PUFA	SFA	χ^2^	*P*-value
Number of pregnancies	10	8	10	0.286	0.867
Total offspring number	33	28	24	1.435	0.488
Total sex ratio	19 ♂ : 14 ♀	18 ♂ : 10 ♀	15 ♂ : 9 ♀	0.312	0.856
Alive : Dead ratio	24 A : 9 D	25 A : 3 D	22 A : 2 D	4.628	0.099
Alive : Dead ratio ♂	14 A : 5 D	16 A : 2 D	13 A : 2 D	1.726	0.422
Alive : Dead ratio ♀	10 A : 4 D	9 A : 1 D	9 A : 0 D	3.775	0.152

### Reproductive parameters

A significant difference among groups was detected in the litter size (*χ* ^*2*^ = 6.855, *P* = 0.032) and the total litter mass (*F*_2,25_ = 4.032, *P* = 0.030), while the sex ratio per litter (*χ* ^*2*^ = 0.311, *P* = 0.856), the gestation duration (*χ* ^*2*^ = 0.573, *P* = 0.751), the individual birth mass (corrected for the pups’ relatedness: *F*_2,25_ = 1.568, *P* = 0.228), and the survival rate at birth (*χ* ^*2*^ = 4.548, *P* = 0.103) did not differ (Table 4). A higher litter size was found in PUFA females compared to SFA females and, correspondingly, a lower total litter mass in the SFA group compared to the control group. Although single pup’s birth mass did not differ among groups, males were barely heavier at birth than females (*F*_1,56_ = 4.059, *P* = 0.049; males: 110.5 ± 2.6 g; females: 105.9 ± 2.8 g).

**Table 4:** Reproductive performance of female guinea pigs maintained on a control (*n* =10), high-PUFA (*n* = 8), or high-SFA (*n* = 10) diet

Reproductive parameter	Control	PUFA	SFA
Litter size^1^	3 (2–5)^ab^	3.5 (2–5)^a^	3 (1–3)^b^
Sex ratio^2^	0.56 (0.36, 0.76)	0.66 (0.44, 0.89)	0.68 (0.49, 0.88)
Gestation duration^1^	69 (67–70)	68 (64–69)	68 (67–73)
Total litter mass^3^	368.8 ± 29.1^a^	355.6 ± 32.5^ab^	260.6 ± 29.1^b^
Individual birth mass^3^	112.1 ± 3.8	102.3 ± 4.2	110.5 ± 4.0
Survival rate^2^	0.73 (0.47, 0.89)	0.89 (0.60, 0.98)	0.92 (0.58, 0.99)

^1^Data given as median and range; analysis via Kruskal-Wallis test

^2^Data given as (mean) effect size and lower and upper confidence limits; analysis via GLM with binomial link

^3^Data given as mean ± s.e.m; analysis via ANOVA or LME

Different superscript letters indicate significant differences between single groups (*P* ≤ 0.05)

Including litter size as covariate in the respective analyses revealed that neither the sex ratio (*χ* ^*2*^ = 0.080, *P* = 0.777) nor the gestation duration (*F*_1,25_ = 0.871, *P* = 0.360) was affected by litter size at all. However, the litter size positively affected the total litter mass (*F*_1,24_ = 145.113, *P* < 0.001) and had a negative effect on the survival rate at birth (*χ* ^*2*^ = 7.706, *P* = 0.006), while only a negative tendency was detected regarding the individual birth mass (*F*_1,26_ = 3.701, *P* = 0.065) (Table 5). Therefore, pups born in larger litters were of lower birth mass and faced a higher mortality rate at birth. Although all these influences did not differ among groups (for all reproductive parameters: *P* > 0.240), the negative effect of litter size on the individual birth mass was only significant in the SFA group, while a significant negative effect of litter size on the survival rate was only detected in the control group (Table 5).

**Table 5:** Effect of litter size on the reproductive performance of female guinea pigs maintained on a control (*n* =10), high-PUFA (*n* = 8), or high-SFA (*n* = 10) diet and the general effect of litter size

Reproductive parameter	Control	PUFA	SFA	Litter size^a^
Sex ratio^b^	0.081 (−0.192, 0.356)	−0.195 (−0.638, − 0.055)	−0.042 (−0.398, 0.170)	−0.017 (−0.016, −0.020)
Gestation duration^c^	0.278 ± 0.675	−0.500 ± 0.954	−0.281 ± 0.939	−0.294 ± 0.315
Total litter mass^c^	110.590 ± 14.616***	90.917 ± 20.756***	95.406 ± 20.426***	98.957 ± 8.215***
Individual birth mass^c^	−2.124 ± 4.669	−4.144 ± 6.664	−10.351 ± 6.950*	−4.929 ± 2.562
Survival rate^b^	−0.174 (−0.382, −0.034)*	−0.035 (−0.460, 0.036)	−0.056 (−0.803, −0.028)	−0.120 (−0.020, −0.273)*

*p ≤ 0.05, **p ≤ 0.01, ***p ≤ 0.001 for significant effects of litter size

^a^Effect of litter size determined after removal of non-significant effects based on the AIC. No significant differences between single groups (for all pairwise comparisons *P* > 0.05)

^b^Data given as (mean) effect size and lower and upper confidence limits; analysis via GLM with binomial link

^c^Data given as slope ± standard error of the slope; analysis via linear model or LME

### Body mass changes in mothers

**Fig. 1:**
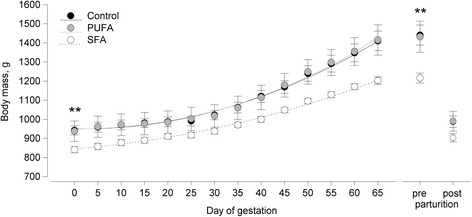
Mean body mass in female guinea pigs maintained on a control, high-PUFA, or high-SFA diet during gestation and after parturition. Circles represent the body mass for each group and day (mean ± s.e.m.); lines for gestation (day 0 to 65) represent the mean fitted values of a linear mixed effect model (corrected for repeated measurements) on the body mass change, including the second-order polynomial term for day and the total litter mass as covariate. Sample sizes: control *n* = 10, PUFA *n* = 8, SFA *n* = 10. ** *P* ≤ 0.01 comparing SFA and the remaining groups. Group:day effect during gestation: *P* ≤ 0.05

**Fig. 2:**
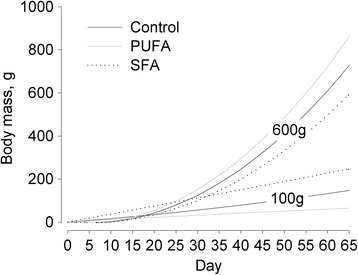
Effect of total litter mass on the body mass gain during gestation in guinea pig females maintained on a control, high-PUFA, or high-SFA diet. Effects were extracted from a linear mixed effect model and are shown for a total litter mass of 100 g and 600 g, respectively. Sample sizes: control *n* = 10, PUFA *n* = 8, SFA *n* = 10. Group:day:total litter mass: *p* ≤ 0.05

From conception until d 65 of gestation (prior to parturition) the body mass increase in mothers differed significantly among groups (group: *F*_2,22_ = 0.992, *P* = 0.387; day: *F*_2,379_ = 0.611, *P* = 0.544; total litter mass: *F*_1,22_ = 2.774, *P* = 0.110; group:day: *F*_4,379_ = 3.296, *P* = 0.011; group:total litter mass: *F*_2,22_ = 2.169, *P* = 0.138; day:total litter mass: *F*_2,379_ = 28.845, *P* < 0.001; group:day:total litter mass: *F*_4,379_ = 3.337, *P* = 0.011). The body mass increase in 5-d intervals was generally less pronounced in SFA females compared to control and PUFA females (control-PUFA day^1^: *t =* 1.715, *P* = 0.087; day^2^: *t* = 0.567, *P* = 0.571; control-SFA day^1^: *t* = 1.975, *P* = 0.049; day^2^: *t* = 0.349, *P* = 0.728; PUFA-SFA day^1^: *t* = 3.433, *P* = 0.001; day^2^: *t* = 0.862, *P* = 0.389) (Fig. 1). Statistical modeling and effect plotting revealed that PUFA females showed the strongest increase in body mass as total litter mass increased, whereas this effect was least pronounced in SFA females (Fig. 2).

**Fig. 3:**
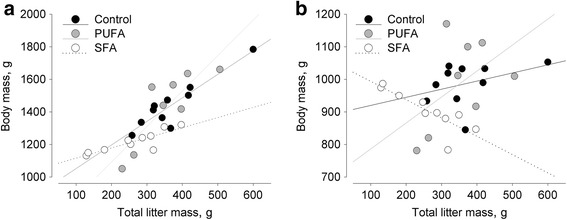
Effects of total litter mass on body mass in guinea pig females maintained on a control, high-PUFA, or high-SFA diet. Effects are corrected for the body mass at conception. **a** Effects on body mass prior to parturition. Control: *p* ≤ 0.001, PUFA: *p* ≤ 0.001, SFA: *p* ≤ 0.001; control vs. PUFA: n.s., control vs. SFA: n.s., PUFA vs. SFA: *p* ≤ 0.05. **b** Effects on body mass after parturition. Control: n.s., PUFA: *p* ≤ 0.05, SFA: n.s.; all group comparisons: n.s. (n.s.: not significant)

On the last day of gestation, before parturition, body mass still differed among groups (*F*_2,25_ = 5.759, *P* = 0.009), with SFA females exhibiting a significantly lower body mass compared to the other groups (control: 1441 ± 52 g; PUFA: 1432 ± 82 g; SFA: 1216 ± 26 g; control-PUFA: *χ* ^*2*^ = 0.013, *P* = 1.000; control-SFA: *χ* ^*2*^ = 9.276, *P* = 0.016; PUFA-SFA: *χ* ^*2*^ = 7.599, *P* = 0.032) (Fig. 1). Corrected for the body mass at conception, the effect of total litter mass on the mother’s body mass prior to parturition differed among groups (body mass at conception: *F*_1,21_ = 38.006, *P* < 0.001; group: *F*_2,21_ = 3.579, *P* = 0.046; total litter mass: *F*_1,21_ = 39.070, *P* < 0.001; group:total litter mass: *F*_2,21_ = 3.990, *P* = 0.034) and was more pronounced in PUFA females compared to SFA females (control-PUFA: *F* = 2.932, *P* = 0.305; control-SFA: *F* = 1.864, *P* = 0.560; PUFA-SFA: *F* = 7.956, *P* = 0.030) (Fig. 3a). The effect of mother’s body mass at conception did not differ among groups and was removed from the model beforehand (group:body mass at conception: *F*_2,19_ = 0.258, *P* = 0.776).

On the first day after parturition, no differences in body mass were detected among the three groups (*F*_2,25_ = 2.134, *P* = 0.140) (Fig. 1). However, compared to the body mass at conception, all groups were significantly heavier after parturition (control: +44.7 g, *t =* 2.405, *p =* 0.024; PUFA: +52.38 g, *t* = 2.521, *P* = 0.018; SFA: +62.8 g; *t* = 3.379, *P* = 0.002). This increase was similar for all groups (*F*_2,25_ = 0.239, *P* = 0.789). Further analyses revealed that the body mass after parturition, corrected for the body mass at conception, tended to be differently affected by the total litter mass (body mass at conception: *F*_1,21_ = 48.454, *P* < 0.001; group: *F*_2,21_ = 3.118, *P* = 0.065; total litter mass: *F*_1,21_ = 0.445; *P* = 0.512; group:total litter mass: *F*_2,21_ = 3.167, *P* = 0.063). While a positive effect of total litter mass on the body mass after parturition was detected for PUFA females (*F* = 4.708, *P* = 0.042), no effects were detected for control and SFA females (control: *F* = 0.445, *P* = 0.512; SFA: *F* = 1.915, *P* = 0.181) (Fig. 3b).

The total body mass loss after parturition was not identical to the total litter mass and therefore the difference probably represented placental tissues and amniotic fluid. This part of the females’ body mass loss differed significantly among the groups (*F*_2,25_ = 3.444, *P* = 0.048) and tended to be lower in SFA females (control: 85 ± 10 g; PUFA: 86 ± 14 g; SFA: 52 ± 9 g; post-hoc analyses with Bonferroni corrections remained non-significant). This, however, was positively affected by the total litter mass, which also diminished the group difference (group: *F*_2,22_ = 0.914, *P* = 0.416; total litter mass: *F*_1,26_ = 33.442, *P* < 0.001; group:total litter mass: *F*_2,22_ = 1.258, *P* = 0.304).

## Discussion

A mother’s dietary fatty acid intake can profoundly affect the reproductive success and may further determine the post-natal development of her offspring. These effects are suggested to be caused by physiological influences of these nutrients already prior to conception, but even by modulating the processes of gestation and parturition [36–38]. Here we could show that diets high in PUFAs and SFAs differently affect reproductive performances in female guinea pigs. These influences, however, were not related to differences in saliva cortisol and plasma estrogen levels at the time of conception among the dietary groups in the present study. Especially PUFAs can positively affect the endocrine system, particularly the hypothalamic-pituitary-adrenal and -gonadal axes and related cortisol, estrogen, and progesterone secretion rates, whereas EPA and AA are also precursors of prostaglandins. All these hormones interactively modulate reproductive processes, including the estrus cycle, conception, the process of gestation, and even parturition [3, 39]. Although we found no differences in cortisol and estrogen concentrations, differences in the analyzed plasma fatty acids indicate modified availabilities of precursors for prostaglandin synthesis, cell membrane composition, and the energy balance.

The dietary supplementations significantly affected the plasma fatty acid status, determined by the percentage of single fatty acids, and, presumably, the availability of these molecules for metabolic functions. Supplementations with walnut oil (high in PUFAs) significantly decreased the percentage of SFAs and increased n-3 and n-6 PUFAs, resulting in the highest P:S ratios. Supplementations with coconut fat (high in SFAs) significantly increased the percentage of SFAs and decreased n-3 PUFAs, resulting in higher n-6:n-3 ratios in these animals. PUFAs and SFAs are seemingly metabolized in different ways: PUFAs are much faster oxidized and SFAs rather stored in abdominal fat [11, 40]. A higher P:S ratio may therefore contribute to the short term energy supply, which would be required for maintaining daily physiological functions, while a lower P:S ratio may indicate that more fat is stored. Hence, the significantly decreased body mass in SFA females at conception seems to be a contradiction, because a higher body fat accumulation could be assumed to result in a higher body mass. However, previous findings in guinea pigs indicate that a lower body mass can also be related to a higher percentage of body fat, although this was found in fetuses [41]. Energetic needs in SFA females were possibly not covered by direct energy supplies via dietary PUFAs but rather via internal energy reserves and perhaps resulted in a reduced body mass. In relation to a lower n-3 PUFA status, this may have negatively influenced the reproductive output in SFA females, because especially n-3 PUFAs are suggested to promote reproductive functions [38]. However, as supplemented females did not exhibit a higher body mass at conception compared to control females, the ad libitum feeding of guinea pig pellets definitely counteracted a possible energetic imbalance among the dietary groups.

Dietary fatty acids differently affected the reproductive output in the studied animals and resulted in a relatively low litter size and total birth mass in the SFA group, which reduced the gestational effort for mothers in the following, as shown by the lowest body mass increase. The majority of SFA females gave birth to a maximum of three pups, whereas control and PUFA females had litter sizes of up to five pups. Ovulation rates and the number of produced and released ova may determine the litter size in rodents and can be highly affected by dietary PUFAs, with n-3 enhancing and n-6 possibly decreasing the number of ova and pups per litter [4, 42]. The increased plasma n-6:n-3 ratio in SFA females may have caused a lower ova production and decreased litter sizes. A possibly lower energy supply by dietary SFA intakes [11, 40] could have negatively affected the production of ova too, in contrast to higher P:S and lower n-6:n-3 ratios as found in PUFA females.

Unbalanced energy intakes in guinea pigs, including food restriction and overfeeding, can generally impact on reproduction and result in decreased litter sizes and individual birth mass [41, 43]. Both diets high in PUFAs and SFAs can diminish these effects, resulting in an increased offspring body mass [6, 8]. Interestingly, no differences were found in the individual birth mass among the dietary treatments in the present study, although birth mass tended to decrease with litter size. Guinea pig pups born in larger litters usually show a lower body mass and also a higher mortality rate [13]. We did not detect such differences among the dietary groups, but a lower pup survival rate at birth was found in larger litters of control females. The fact that even dead-born pups were fully developed and still covered in their amniotic sacs indicates that they died at birth, probably because mothers did not rupture the amniotic sacs and the pups therefore asphyxiated soon after birth [44]. Mothers can be assumed to be exhausted by giving birth especially to large litters, whereas dietary fatty acids may provide additional energy during the process of parturition, thereby facilitating maternal care for the neonates, or even affect the morphology of the birth canal, resulting in an easier parturition. A higher energy intake and the resulting improved body condition of a mother may also favor the birth of male offspring [45], but we found no differences in the sex ratio at birth or in the ratio of dead and alive born male and female pups. These results are in contrast to a variety of mechanisms describing how diet and available energy may skew the sex ratio [46], which has also been reported for high-fat diets [9]. The present results suggest that guinea pigs generally produce more male offspring, which is apparently not affected by dietary fat intakes. As relatively low mortality rates and sex ratios higher than 0.5 at birth were detected in all dietary groups, it can be concluded that females used in this study were in good body conditions.

An increased survival rate in large litters is not only of major importance regarding the lifetime reproductive success, but also in terms of a mother’s investment during gestation, as reflected in the relative body mass increase during gestation caused by the developing fetuses. A very high body mass increase during gestation represents a significant energetic effort in guinea pigs especially during the second half of gestation [47]. As the litter size and total birth mass in SFA mothers were generally lower, at least compared to PUFA females, this obviously resulted in a reduced gestational effort in these animals. PUFA females, in contrast, showed highest gestational efforts and were seemingly able to support the developing pups especially in case of larger litters by an increased energetic investment during gestation, which resulted in a similar individual birth mass among different litter sizes and groups. An increased energy allocation in the developing fetuses could have also eliminated a negative relationship between litter size and individual birth mass, which usually occurs in guinea pigs [13, 14]. The relatively low litter sizes and total litter masses in SFA females, which resulted in the low gestational efforts, perhaps enabled these animals to provide the developing fetuses with adequate energy, which might have not been possible in case of larger litters.

All females revealed a significantly increased body mass after parturition compared to conception, indicating available internal energy reserves for the lactation period after the exhausting parturition. Although female guinea pigs energetically invest more in their offspring during gestation compared to lactation [16], the short lactation period is usually characterized by a regular decrease in the mothers’ body mass [26]. SFA females in the present study showed the most pronounced body mass gain from conception to post parturition. This may indicate that these animals were able to accumulate higher internal energy reserves during gestation, perhaps due to the lower number of developing fetuses and a related lower energetic investment. PUFA females, however, seemed to adjust their own body mass to the litter size and total litter mass, as the total litter mass positively affected the mother’s body mass after parturition. This was not detected in control and SFA females and also a previous study in guinea pigs did not reveal such an effect [43]. However, a higher gestational effort, litter size, and total litter mass could be assumed to impair a mother’s body condition after parturition. As this is apparently not the case in guinea pigs, it can be concluded that these animals are able to accumulate additional energy reserves during gestation, which may be enhanced by feeding on dietary PUFAs. A lower litter size and total litter mass in SFA females may have further been related to less pronounced placental tissues [48], which perhaps also caused a decreased energy allocation in establishing and maintaining these tissues. This would also be supported by a general positive link to the total litter mass, as more and heavier fetuses require more space in the uterus and have to be provided with more oxygen and nutrients, resulting in larger and heavier placentas [41].

## Conclusions

SFAs seem to reduce the litter size in guinea pigs, which would reduce the lifetime reproductive success probably due to inadequate available energy for reproductive functions and for developing a higher number of fetuses. In this way, SFAs may also decrease the gestational effort, which perhaps favors body condition in these animals during and after gestation due to a lower energy allocation required for the developing fetuses. Dietary PUFAs seem to modify energy accumulations in pregnant guinea pigs in relation to the litter size and total litter mass towards the maximum reproductive success. Although both types of fatty acids seem to affect female reproduction at different levels, they may both maximize the survival rate at birth and therefore the different gestational efforts would be justified. These promising results may further help to understand how dietary fatty acids can modulate reproductive processes and the reproductive success, perhaps by primarily affecting the mother’s body condition. This demonstrates the significant influence of dietary fatty acids on mammalian reproduction, since not only the developing fetuses but also a mother’s reproductive performance may be highly influenced by an adjustment of pre-fertilization dietary PUFA and SFA intakes. However, a limitation of this study is the lack of knowledge which male fertilized the female, because a high rate of multiple paternities could have been possible due to our experimental setup [49]. Paternal effects in relation to dietary fatty acids (e.g. number of mutation rates [50]) should be considered in order to fully interpret the whole impact of these nutrients on reproduction in mammals.

## Additional file

Additional file 1: Dataset.xls; Dataset supporting the results and conclusions of this article. (XLS 56 kb)

## Abbreviations

AA: Arachidonic acid
AIC: Akaike information criterion
ALA: α-linolenic acid
ANOVA: Analysis of variance
DHA: Docosahexaenoic acid
EPA: Eicospentaenoic acid
FAMES: Fatty acid methyl esters
LA: Linoleic acid
LME: Linear mixed effects model
n-3: Omega-3
n-6: Omega-6
PUFA: Polyunsaturated fatty acid
SFA: Saturated fatty acid
